# Evaluation of Analgesic Activity of the Methanol Extract from the Galls of *Quercus infectoria* (Olivier) in Rats

**DOI:** 10.1155/2014/976764

**Published:** 2014-08-31

**Authors:** Sook-Ha Fan, Noraisah Akbar Ali, Dayang Fredalina Basri

**Affiliations:** ^1^Department of Biomedical Science, Faculty of Science, Universiti Tunku Abdul Rahman, Jalan Universiti, Bandar Barat, 31900 Kampar, Perak, Malaysia; ^2^School of Diagnostic and Applied Health Sciences, Faculty of Health Sciences, Universiti Kebangsaan Malaysia, Jalan Raja Muda Abdul Aziz, 50300 Kuala Lumpur, Malaysia

## Abstract

The present study aims to investigate the analgesic activity of the methanol extract of the galls of *Quercus infectoria* in rats using hot plate and tail-flick methods. The extract was administered intraperitoneally at a dose of 20 mg/kg while morphine sulfate and sodium salicylate (10 mg/kg) served as standards. The methanol extract exhibited significant analgesic activity in the tail-flick model (*P* < 0.05) by increasing the reaction time of the rats to 8.0 sec at 30 min after treatment in comparison to control (4.4 sec). Morphine sulfate produced a reaction time of 11.9 sec in the same test. At the peak of activity (30 min), the extract produced maximum possible analgesia (MPA) of 34.2%, whilst morphine sulfate achieved a peak MPA of 70.9%. No analgesic effects have been observed using sodium salicylate in the tail-flick model. In the same model, the extract and sodium salicylate demonstrated comparable reaction times. Tail-flick is a better method to evaluate analgesic activity as no significant results were observed for all treatments using hot plate with the exception of morphine sulfate, which showed significant results only at 45 and 60 min after treatment. In conclusion, the methanol extract of the galls of *Quercus infectoria* displayed analgesic activity.

## 1. Introduction

According to the International Association for the Study of Pain (IASP), pain is defined as “an unpleasant sensory and emotional experience associated with actual or potential tissue damage” [1]. Common drugs for pain relief such as aspirin and morphine have been widely used in recent decades. In most instances, these analgesic drugs, particularly opioids and nonsteroidal anti-inflammatory drugs (NSAIDs), can only relieve 50% of the pain in about 30% of patients [2]. In addition, many of these drugs cause serious side effects. Studies have shown that opiates cause physical dependency, tolerance, and addiction while NSAIDs usually cause gastrointestinal disorders [3].

As such, research to discover other alternatives to treat pain is crucial. Medicinal herbs have been used for centuries for therapeutic purposes. Many of these herbs with analgesic activity had been used without any adverse effects.* Quercus infectoria *(Olivier) from the family Fagaceae is commonly known as gall oak or dyer's oak. The semievergreen, small tree is indigenous to Turkey, Iran, Greece, and Syria. The galls of* Quercus infectoria* are the result of a perforation made in the bark by gall wasps such as* Diplolepis gallae tinctoriae* or* Cynips quercufolii* to deposit their eggs. The galls are locally known as “manjakani” in Malaysia and Indonesia or “majuphal” in India [4].

Studies have shown that the main constituent of the galls are tannin (36–60%), gallic acid, ellagic acid, and syringic acid [5]. Traditionally, the galls have been used to restore postpartum uterine elasticity among the Malays, in the treatment of toothache and gingivitis among the Indians, and as cure for inflammatory diseases in some Asian countries [6]. Pharmacologically, it has been documented to possess antioxidant, anti-inflammatory, antimicrobial, and antidiabetic activities [7]. The present study aimed to evaluate the analgesic activity of methanol extract of galls of* Quercus infectoria* in animal models.

## 2. Materials and Methods

### 2.1. Plant Materials

The galls of* Quercus infectoria* used in this study were purchased from a local market in Kuala Lumpur. The galls were identified and deposited at the Forest Research Institute Malaysia (FRIM) with the voucher number EZ186/93. The dried galls were crushed to small pieces using pestle and mortar and pulverized using an electric grinder.

### 2.2. Preparation of Extract

The methanol extract was prepared by cold extraction technique [8]. About 100 g of the dried gall powder was immersed in 500 mL methanol for 24 h at room temperature. The mixture was then filtered and the process was repeated using the remaining residue with 300 mL methanol. Both filtrates were then combined and concentrated under reduced pressure using rotary evaporator. The resulting semisolid residue was pounded to dryness under hot-air dryer to obtain a powdery crude methanol extract.

### 2.3. Animals

Mature albino Wistar rats (*Rattus norvegicus*) (150–200 g) of both sexes obtained from the Laboratory Animal Units of the Faculty of Medicine, Universiti Kebangsaan Malaysia, were used for the experiment. The animals were housed under standard laboratory conditions at room temperature with relative humidity of 70–80%. They were fed with standard commercial diet and water* ad libitum*. Prior to the experiment, the animals were fasted for 12 h with water given* ad libitum* and weighed. All procedures described were reviewed and approved by the Universiti Kebangsaan Malaysia Animal Ethics Committee (UKMAEC) (UKM 1.5.24/111/61/2).

### 2.4. Study Design

The rats were randomly assigned to four groups of six animals each for the two different experimental models. The first group served as negative control receiving normal saline (10 mL/kg). The second and third groups served as positive control and were given standard drugs, morphine sulfate and sodium salicylate, respectively (10 mg/kg each). The methanol extract of the galls of* Quercus infectoria* was given at a dose of 20 mg/kg to the last group. All treatments were administered intraperitoneally.

### 2.5. Tail-Flick Test

Antinociceptive (analgesic) activity of the extract was evaluated by the tail-flick method described [9]. About 5 cm from the distal end of the tail of each rat was immersed in warm water maintained at 50°C. The reaction time (in seconds) was the time taken by the rat to flick its tail due to pain. The first reading was omitted and reaction time was taken as the average of the next two readings. The reaction time was recorded before (0 min) and at 15, 30, 45, and 60 min after the administration of the treatments. The maximum reaction time was fixed at 15 sec to prevent any tail tissue injury. If the reading exceeds 15 sec, it would be considered as maximum analgesia. The maximum possible analgesia (MPA) was calculated as follows:

(1) MPA=Reaction  time  for  treatment−reaction  time  for  saline15 sec−reaction  time  for  saline ×100.

### 2.6. Hot Plate Test

Evaluation of analgesic activity of the extract was also carried out using hot plate method [10]. The rats were placed on a hot plate maintained at 55°C within the restrainer. The reaction time (in seconds) or latency period was determined as the time taken for the rats to react to the thermal pain by licking their paws or jumping. The reaction time was recorded before (0 min) and at 15, 30, 45, and 60 min after the administration of the treatments. The maximum reaction time was fixed at 45 sec to prevent any injury to the tissues of the paws. If the reading exceeds 45 sec, it would be considered as maximum analgesia. The maximum possible analgesia (MPA) was calculated as follows:

(2) MPA=Reaction  time  for  treatment−reaction  time  for  saline45 sec−reaction  time  for  saline ×100.

### 2.7. Statistical Analysis

Data were presented as mean ± standard error mean (SEM). The results were analyzed using Statistical Package for the Social Sciences (SPSS) version 16. Statistical significance was determined by Student's *t*-test and *P* value less than 0.05 was considered as significant.

## 3. Results

### 3.1. Tail-Flick Test

The results of the analgesic activity of the methanol extract of the galls of* Quercus infectoria* are shown in Table 1. Rats treated with normal saline (negative control) did not show any significant difference in the reaction time on tail-flick throughout the 60 min observation. In comparison with the baseline values within the same treatment groups, the increase in reaction time at different time points significantly differed (*P* < 0.05) for morphine sulfate only. Duration of the reaction time in morphine sulfate and extract treated animals was significantly higher compared to saline treated animals, except for the extract group at 60 min. The highest reaction time for the extract treated group was 8.0 sec at 30 min, while it was 4.4 sec and 11.9 sec for saline and morphine sulfate groups, respectively. At all time points, the tail-flick latency time differed significantly between the extract and morphine sulfate groups, being greater for the latter group. No significant difference in reaction time was observed between the extract and sodium salicylate. Observation in rats treated with sodium salicylate did not give any significant analgesic effect in comparison with baseline values, saline, or extract (except for 30 min after treatment).

**Figure 1:**
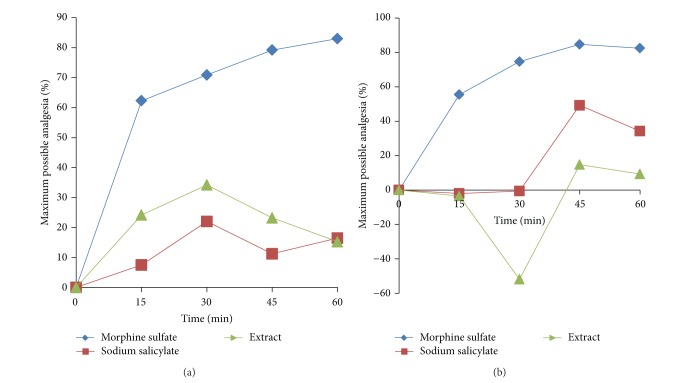
Maximum possible analgesia (MPA) (%) representing the effect of the methanol extract of the galls of* Quercus infectoria* compared to morphine sulfate and sodium salicylate (positive control) administered into rats, evaluated by (a) tail-flick method and (b) hot plate method.

The analgesic effects of morphine sulfate, sodium salicylate, and extract could be seen from the maximum possible analgesia (MPA) graph (Figure 1(a)). The analgesic effect of morphine sulfate was evident within 15 min following intraperitoneal administration. The MPA remained elevated during the observation period, reaching its peak at 60 min (83.0%). Likewise, the extract also showed analgesic activity beginning at 15 min, with the highest MPA at 30 min, and gradually decreased towards 60 min (34.2%). For sodium salicylate, the MPA exhibited similar trend, producing a peak at the same time point (22.0%). With reference to MPA value, the extract demonstrated stronger analgesic activity than sodium salicylate at all time points.

### 3.2. Hot Plate Test

The results of the analgesic effect of the methanol extract of the galls of* Quercus infectoria* using hot plate method are presented in Table 2. The results showed that there was no significant difference on the thermal stimulus in rats treated with normal saline (negative control) throughout the 60 min observation. There was no increase in reaction time at all time points compared to baseline values (0 min) within the same treatment groups. In comparison to the saline treated animals, the significant increase in the reaction time to thermal pain was not detectable in both sodium salicylate and extract with the exception of morphine sulfate. However, the observation in morphine sulfate treated animals is only noted at 45 and 60 min. The reaction time was significantly different between the extract and morphine sulfate, being greater for morphine sulfate at 30, 45, and 60 min after treatment. No significant difference was observed between the extract and sodium salicylate.

Figure 1(b) illustrates the analgesic effect of morphine, sodium salicylate, and extract using MPA. Morphine sulfate elicited significant analgesic activity within 15 min following administration as evidenced by the gradual increase throughout the observation period. At the peak of activity (45 min), morphine sulfate showed MPA of 84.7%. Rats treated with sodium salicylate exhibited analgesic activity at a slower interval, which began at 45 min (49.2%) and then declined. The MPA value for the extract did not show any analgesic effect in the first 30 min after treatment but increased at 45 min (14.8%) and declined thereafter.

On the basis of these findings, tail-flick is a better method to evaluate analgesic activity compared to hot plate as no significant results were observed for all treatments using hot plate with the exception of morphine sulfate.

## 4. Discussion

Analgesics are drugs that act on peripheral or central nervous system to selectively relieve pain without significantly altering consciousness [11]. Centrally acting analgesics act by raising the threshold for pain and also altering the physiological response to pain. On the other hand, peripherally acting analgesics act by inhibiting the generation of impulses at chemoreceptor site of pain [12]. The animal models employed for screening of analgesic activity in this study are pain-state models using thermal stimuli which include tail-flick and hot plate methods. Both methods are useful in illustrating centrally mediated antinociceptive responses which focus generally on changes above the spinal cord level [13]. While the tail-flick method mediates a spinal reflex to a nociceptive stimulus, hot plate method involves higher brain functions and is regarded a supraspinally organized response [14].

In tail-flick model, the methanol extract from the galls of* Quercus infectoria* exhibited significant analgesic activity by increasing the reaction time of the rats compared to control (saline treated rats) at all time points, except at 60 min. Sodium salicylate and morphine sulfate were used as reference drugs, which are considered mild and moderate to severe analgesics, respectively. In comparison with control, morphine produced the most significant antinociception effect during all observation times, followed by the extract, while no significant analgesic effect was observed for sodium salicylate. The tail-flick method is based on the observation that morphine-like compounds are selectively able to prolong the reaction time of typical tail-withdrawal effect in rats. This method is also useful in differentiating central opioid-like analgesics from peripheral analgesics [15]. Analgesic drugs which are centrally acting elevate pain threshold of animals towards heat and pressure [16]. Therefore, the analgesic effect of the extract on this pain-state model indicates that it might be centrally acting. With reference to the MPA value, the analgesic effects of both the extract and morphine sulfate were evident within 15 min following intraperitoneal administration. However, the extract showed short-lived analgesia as the MPA gradually decreased after 30 min compared to morphine sulfate. The tail-flick latency of the extract at all time points was less than that of reference drug, morphine sulfate, which is a slow onset opioid with long duration of action [17]. Although there was no significant analgesic effect between the reaction time of the extract and sodium salicylate, the extract exhibited a non-significant trend of higher reaction time compared to sodium salicylate. Both treatments produced comparable reaction times, suggesting that the galls of* Quercus infectoria* could be a better natural alternative for mild pain relief.

The methanol extract from the galls of* Quercus infectoria* failed to increase the reaction time of the rats on hot plate method in this study. The difference in the mean reaction time of the extract and the control groups was not statistically significant during all observation times. Analgesia in morphine sulfate treated rats was only detectable at 45 and 60 min. No significant analgesic effect was observed between sodium salicylate and control and the extract tested. Hot plate method produces two measureable behavioural components in response to thermal pain, with regard to their reaction times. Responses such as paw licking and jumping in rats are considered to be supraspinally integrated [15]. Thus, the failure of the extract to inhibit these behaviours on hot plate method indicates that it might not be acting at supraspinal level.

Taken together, tail-flick is a better method to evaluate analgesic activity compared to hot plate as no significant results were observed for all treatments using hot plate with the exception of morphine sulfate. This observation is in agreement with findings of [18] which reported that the extract of* Clerodendrum phlomidis* produced a significant elongation of the tail-flick reaction time but not in hot plate method.

Tail-flick and hot plate are two of the several methods available for evaluating central analgesic activity [15]. Although both methods employed thermal stimuli, the tail-flick response indicates spinally mediated reflex while the paw-licking hot plate response is due to complex supraspinally integrated behaviour [14]. Findings from this study demonstrated that the methanol extract prolonged the reaction time in the tail-flick method but showed an apparent lack of effect in the hot plate method. This might indicate higher sensitivity of the spinally mediated reflex response in the tail-flick method. However, intra-animal variation may also contribute to the lack of effect in hot plate method. Unlike the typical tail-withdrawal reflex in rats, problem arises in the hot plate method as the rats have to learn what nociceptive response they need to show in order to stop the thermal stimulus [19]. Taken together, the differences in sensitivity of both methods as well as the mechanism involved may explain the analgesic effects observed in this study.

A number of alkaloids, flavanoids, steroids, and tannin isolated from medicinal plants have been reported to possess significant analgesic activity [20]. The major constituent of the galls from* Quercus infectoria* is tannin, which comprises up to 60% of its total content [5]. Thus, analgesic activity observed with this extract might be attributed to the presence of this compound. Furthermore, there are reports on the role of tannin in analgesic activity. According to [21], preliminary phytochemicals which were screened from* Scoparia dulcis* L. including tannin might be responsible for the observed analgesic activity. Another research suggested that the presence of tannin and flavonoid in the methanol extract of* Cassia auriculata* leaves seems to inhibit prostaglandin synthesis and exerts the anti-inflammatory and analgesic effects [22].

This preliminary study did not fully demonstrate the dose-dependent analgesic effect of the extract of the galls of* Quercus infectoria* because different concentrations of the extract were not tested, which remains a limitation of the present study. Future investigations might be directed to examine if the analgesic activity was related to the dose and other animals models such as carrageenan-induced paw oedema to test for anti-inflammatory activity and acetic acid-induced abdominal contraction test or writhing assay for the evaluation of peripheral antinociceptive activity.

## 5. Conclusion

In conclusion, the methanol extract of the galls of* Quercus infectoria* displayed analgesic activity and supported the traditional use of this plant in pain relief. Further study is warranted to identify the active compounds present in this extract and to elucidate the mechanisms involved in its analgesic properties.

## Figures and Tables

**Table 1:** Analgesic effect of methanol extract from the galls of *Quercus infectoria* by tail-flick method in rats.

Treatments	Reaction time in seconds (mean ± SEM)				
	0 min	15 min	30 min	45 min	60 min
Control (normal saline)	4.25 ± 0.57	4.50 ± 0.34	4.42 ± 0.45	4.58 ± 0.44	5.17 ± 0.80
Morphine sulfate	6.50 ± 1.22	11.04 ± 0.73^∗ab^	11.92 ± 0.84^∗ab^	12.83 ± 0.35^∗ab^	13.33 ± 0.83^∗ab^
Sodium salicylate	4.13 ± 0.54	5.29 ± 0.57	6.75 ± 0.62^∗a^	5.75 ± 0.56	6.79 ± 1.24
*Quercus infectoria *(galls) extract	6.67 ± 0.85^a^	7.04 ± 0.67^a^	8.04 ± 0.73^a^	7.00 ± 0.92^a^	6.67 ± 0.86

All values by Student's *t*-test, significant at *P* < 0.05, and SEM = standard error mean. *P < 0.05 versus baseline of the respective treatment, ^a^ *P* < 0.05 treatment versus control, ^b^ *P* < 0.05 extract versus morphine sulfate, extract versus sodium salicylate was not significant at all time points.

**Table 2:** Analgesic effect of methanol extract from the galls of *Quercus infectoria *by hot plate method in rats.

Treatments	Reaction time in seconds (mean ± SEM)				
	0 min	15 min	30 min	45 min	60 min
Control (normal saline)	30.67 ± 5.15	28.25 ± 4.87	31.50 ± 4.57	24.08 ± 6.37	24.83 ± 6.02
Morphine sulfate	30.75 ± 5.64	37.54 ± 5.55	41.58 ± 3.22^b^	41.79 ± 2.87^ab^	41.46 ± 2.55^ab^
Sodium salicylate	32.04 ± 4.59	27.92 ± 4.04	31.42 ± 2.61	34.38 ± 2.08	31.73 ± 1.46
*Quercus infectoria *(galls) extract	30.88 ± 4.08	27.67 ± 2.17	24.50 ± 3.08	27.17 ± 3.92	26.71 ± 4.06

All values by Student's *t*-test, significant at *P* < 0.05, and SEM = standard error mean. ^a^ *P* < 0.05 treatment versus control, ^b^ *P* < 0.05 extract versus morphine sulfate, The baseline values versus other time points within the same treatment groups were not significant. Extract versus sodium salicylate was also not significant at all time points.
